# Coordinated Evolution of Transcriptional and Post-Transcriptional Regulation for Mitochondrial Functions in Yeast Strains

**DOI:** 10.1371/journal.pone.0153523

**Published:** 2016-04-14

**Authors:** Xuepeng Sun, Zhe Wang, Xiaoxian Guo, Hongye Li, Zhenglong Gu

**Affiliations:** 1 Division of Nutritional Sciences, Cornell University, Ithaca, New York 14853, United States of America; 2 College of Agriculture and Biotechnology, Zhejiang University, Hangzhou 310058, P.R. China; University of Strasbourg, FRANCE

## Abstract

Evolution of gene regulation has been proposed to play an important role in environmental adaptation. Exploring mechanisms underlying coordinated evolutionary changes at various levels of gene regulation could shed new light on how organism adapt in nature. In this study, we focused on regulatory differences between a laboratory *Saccharomyces cerevisiae* strain BY4742 and a pathogenic *S*. *cerevisiae* strain, YJM789. The two strains diverge in many features, including growth rate, morphology, high temperature tolerance, and pathogenicity. Our RNA-Seq and ribosomal footprint profiling data showed that gene expression differences are pervasive, and genes functioning in mitochondria are mostly divergent between the two strains at both transcriptional and translational levels. Combining functional genomics data from other yeast strains, we further demonstrated that significant divergence of expression for genes functioning in the electron transport chain (ETC) was likely caused by differential expression of a transcriptional factor, *HAP4*, and that post-transcriptional regulation mediated by an RNA-binding protein, *PUF3*, likely led to expression divergence for genes involved in mitochondrial translation. We also explored mito-nuclear interactions via mitochondrial DNA replacement between strains. Although the two mitochondrial genomes harbor substantial sequence divergence, neither growth nor gene expression were affected by mitochondrial DNA replacement in both fermentative and respiratory growth media, indicating compatible mitochondrial and nuclear genomes between these two strains in the tested conditions. Collectively, we used mitochondrial functions as an example to demonstrate for the first time that evolution at both transcriptional and post-transcriptional levels could lead to coordinated regulatory changes underlying strain specific functional variations.

## Introduction

It has been well established that gene regulation plays a critical role in environmental adaptation [1–3]. In the past decades, transcriptional regulation has been the most studied field for the evolution of gene regulation in numerous species. Organisms show a remarkable regulatory flexibility that allows them to thrive under diverse external conditions, and this plasticity could be achieved by gene regulation at different levels [4]. With new strides in technology, the evolution of gene regulation at levels other than transcription has been investigated [5–9]. Exploring coordinated evolutionary changes for gene regulation could shed new light on how organism adapts in nature.

Yeasts offer unique advantages for both evolutionary and functional research. More than a thousand different yeast species have now been identified [10]. Yeasts are found in a wide range of environments with distinctive phenotypes. The genotypic changes underlying phenotypic diversity are yet to be investigated for most species except for the *Saccharomyces sensu stricto* species complex, where a large spectrum of genetic variation has been extensively studied, including chromatin structure [11], genomic features [12], gene transcription and translation [7, 13], and protein abundance [14]. In budding yeast *S*. *cerevisiae*, strains show significantly different phenotypes, including growth rate, ethanol production, morphology and pathogenicity [15, 16]. Previous work on deciphering genetic variations in different populations has focused on the layers of nucleotide polymorphism [15, 17, 18], gene transcription [19, 20] and protein/metabolite abundance [19], whereas the regulatory differences within post-transcriptional and translational levels are still largely unclear.

It has been recently shown that gene expression diverged most dramatically among yeast strains in their mitochondrial functions [19]. The mechanisms underlying this observation remain unknown. Furthermore, the mitochondrion is the major source of cellular energy. It is also the center for biosynthesis of many metabolites and bioactive products that could regulate other cellular processes [21, 22]. A dysregulation of mitochondrial function may have a broad impact on genome stability and gene expression [21]. The closely interacting protein complexes that function in mitochondria often impose tight coevolution [23]. On the other hand, due to high mutation rate in the mitochondrial genome [24, 25], genes encoded by mtDNA evolve at a different rate from the genes encoded by nuclear genome, which could lead to incompatible mitochondrial and nuclear genomes. Indeed, an interrupted mito-nuclear interaction has been observed to cause severe impacts on organism viability [26] or reproduction [27]. Interestingly, a recent study indicates that mito-nuclear genome combination between yeast strains could also provide growth advantages for certain backgrounds under specific growth conditions [28], implying the complex nature of mito-nuclear epistasis.

In this study we focused on gene regulation of mitochondrial function and interaction between the mitochondrial and nuclear genomes for a laboratory *S*. *cerevisiae* strain BY4742 (derived from strain S288c) and YJM789, a pathogenic *S*. *cerevisiae* strain derived from haploid progeny of YJM145 that was initially isolated from an AIDS patient [29]. YJM789 differs from the laboratory strain in many aspects, such as high temperature tolerance, growth rate under normal conditions, and colony morphology [30, 31]. Mating between YJM789 and S288c reduces spore viability when compared with mating within the S288c backgrounds [32]. The YJM789 genome is remarkably different from S288c with ~60,000 SNPs and ~6,000 indels, in addition to multiple ORFs that are unique to YJM789 [30]. Genetic variations responsible for high temperature growth in YJM789 have been identified at nucleotide resolution [31], however, the effects of these quantitative trait genes are strain dependent [33]. Previous studies that compared between YJM789 and laboratory strains were mainly focused on pre-transcription/transcription level divergence [16, 30, 34], whereas the post-transcriptional regulation is under-investigated. In this study, we profiled the transcriptome (through RNA-Seq, hereafter designated as mRNA) and translatome (through ribosomal footprint profiling, hereafter designated as RFP) of YJM789 and BY4742 to compare their regulation at both levels simultaneously. We also constructed a hybrid strain combining nuclear and mitochondrial genomes from the two strains, and studied the influence of mito-nuclear interactions on growth and nuclear gene expression.

## Materials and Methods

### Yeast strains and growth condition

The *S*. *cerevisiae* laboratory strain BY4742 (MATα, *hoD*::*NatMX*, *his3*, *lys2*, *trp*, *ura3*), the clinical strain YJM789 (MAT-α, *ho*::*hisG*, *lys2*, *gal2*) and the *KAR1-1* strain MCC123 rho^0^ (MATa, *ade2-101*, *ura3-52*, *kar1-1*) were used in this study. All strains and replicates were cultured in YPD at 30°C, 200 rpm. We conducted at least two replicates for all experiments throughout the study.

### Sequencing library preparation of mRNA and RFP

Both strains were cultured in YPD medium starting at OD_600_ = 0.1, and harvested when OD_600_ reached 0.6 ~ 0.8. Cells were then immediately treated with 100 μg/ml cycloheximide (CHX) for 2 minutes to inhibit cytosolic translation elongation. The treated cells were divided into two fractions for mRNA and ribosomal footprint library preparation. To construct the mRNA sequencing library from one fraction, total RNA was extracted using the hot phenol method [35], mRNA was purified using oligo-dT DynaBeads (Life Technologies Co., USA), and the cDNA library was constructed using the method described by Zhong *et al*. [36]. The other fraction of cells was ground in a mortar with liquid nitrogen. Ribosomal polysomes were extracted from the aliquots, and footprint mRNA was then released from polysomes for sequencing library preparation, following the method described by Wang *et al*. [37]. All libraries were submitted to Illumina HiSeq 2000 platform for deep sequencing (50bp, single-end).

### Modification of genome annotation

To refine the genome annotation of YJM789, we predicted *de novo* gene models by feeding the overall mRNA data to AGUSTUS [38]. The resulted gene models were then compared with the previous annotation using custom perl scripts according to the following criteria: (i) both mRNA and RFP read coverage were required to confirm a new gene prediction. The minimum read count was 50 for both mRNA and RFP, and the putative gene was required to be longer than 150 bp (50 amino acids); (ii) if the new model increased the length of an existing annotation, then mRNA and RFP coverage was required to be ≥ 20 reads, and the presence of the start/stop codon is also required; (iii) if the predicted model shortened an existing annotation, the prediction was retained only if no mRNA and RFP reads were observed on the corresponding region. All refined annotations were manually checked using the integrative genomics viewer (IGV) [39].

### Read mapping and transcription/translation quantification

Raw reads from mRNA and RFP were cleaned by removing the adaptors/barcodes, and filtered by quality using custom perl scripts. The cleaned mRNA reads were mapped to the corresponding genomes using SOAP [40], with no more than two mismatches allowed. For the RFP reads, since the footprinted mRNA fragments were introduced with spurious adenine bases to the 3’ end, it was difficult to distinguish the real 3’-bound of protected footprint fragments [41]. To solve this, an iterative mapping approach was used [41]. Specifically, reads from both strains were iteratively mapped to the corresponding genomes using SOAP after removing the 3’-most nucleotide one at each time, and an alignment was assigned when the truncated read was no smaller than 23 bp and had no more than two mismatches. The uniquely aligned reads from both mRNA and RFP were assigned to genomics features, such as coding sequence (CDS, 16 bases upstream of first nucleotide and 14 bases upstream of last nucleotide), intron (first base to 8 bases upstream of last nucleotide), 5’-UTR (first base to 17 bases upstream of first nucleotide in CDS) or 3’-UTR (13 bases upstream of last nucleotide in CDS and 28 bases upstream of last nucleotide), based on the position of the 5’-most nucleotide [41]. To quantify the mRNA and RFP reads, we first obtained the base level read coverage of all ortholog genes (6,246) from both species. A minimum of 50 reads mapped to the CDS region were required to retain the gene for further transcriptional and translational analysis, resulting in 5,301 genes for mRNA and 3,663 genes for RFP.

The test for identifying statistical significance of mRNA and RFP divergence was adopted from the method by Artieri and Fraser [13] (see results for details). Briefly, we sought to generate a sequence-specific null distribution accounting for the influence of sequencing bias and gene length for both mRNA and RFP. We let L_B_ and L_Y_ denote, respectively, the mappable lengths of BY4742 and YJM789 orthologs, and let π_B_ = [π_B_(A), π_B_(T), π_B_(G), π_B_(C)] and π_Y_ = [π_Y_(A), π_Y_(T), π_Y_(G), π_Y_(C)] denote the corresponding marginal nucleotide frequencies of each ortholog. We began to form the null ortholog pairs by resampling, with replacement, the base level counts from either BY4742 or YJM789 ortholog using the same length and nucleotide frequency from the orthologs (L_B_, π_B_ or L_Y_, π_Y_), The resampling was repeated 10,000 times and the null distribution of the log_2_-transformed ratio of BY4742 to YJM789 was derived from two resampling of both replicates. We compared the observed base level counts log_2_-ratio of BY4742 to YJM789 with the underlying null distributions to obtain two-sided *P* values (four values for each ortholog pair). The maximum *P* value was retained.

To test for statistical significance in translation efficiency (denoted as RFP/mRNA) divergence, we sought to test the null hypothesis that log_2_[RFP (BY4742 /YJM789)] was not significantly different from log_2_[mRNA (BY4742 /YJM789)]. Therefore, we resampled, respectively, L_B_, π_B_ and L_Y_, π_Y_ base level counts from RFP data in BY4742 and YJM789 with replacement 10,000 times. Each resampling was used to generate the null distribution of log_2_[RFP (BY4742 /YJM789)]. We then compared the observed log_2_[mRNA (BY4742/YJM789)] to the permuted RFP distribution to obtain a *P* value. The same resampling was conducted reciprocally in mRNA data, which was compared with observed log_2_[RFP (BY4742 /YJM789)]. The maximum *P* value was retained if the directionality of difference agreed among all comparisons from all replicates. The final *P* values were adjusted to control false discovery rate (FDR) using the method in Benjamini and Hochberg [42]. FDR ≤ 0.05 was used to determine the significance throughout the study.

### GO enrichment analysis and motif investigation

GO enrichment analysis was conducted using the online service DAVID [43]. An FDR-corrected *P* value ≤ 5% was determined as significant enrichment. Motif analysis was implemented using the MEME suite [44]. The 1 kb upstream and downstream sequences of the candidate genes were extracted and fed to MEME for motif searching using the zoo (zero or one) model with up to 50 motifs. The resulted motifs were then submitted to the TOMTOM tool incorporated in the MEME suit for motif annotation.

### Calculating ribosome residence time (RRT) for the 61 sense codons

RRT was calculated using the method from Gardin *et al*. [45]. All reads with length ≥ 27 bp that mapped to an in-frame codon were retained for analysis. For each footprint, there were 9 possible positions (codon positions 1 to 9) where a codon could appear. For each of the 61 sense codons, we identified all 17-codon windows in all coding regions across the genome, in which the codon occurs uniquely in the middle of the window. For each such window, we calculated the total number of footprints for each of the nine positions where the codon could appear, and then divided this by the total number of footprints aligned to this window, to get the relative frequency (RF) of each position. The overall relative frequency was obtained by averaging RF across all windows at the genome level.

In the absence of any codon preference of the ribosome, we would expect a uniform distribution of RF for each codon across all possible 9 positions (11%). However, if the ribosome dwelt for an extended time over any codon, there should be enriched footprint on that position. We calculated RRT for each codon at each of the nine positions by dividing the observed RF by the expected RF at that position. To test for the statistical significance of ribosome stalling, we compared the observed RF to a distribution of 10,000 RFs found by permuting footprint counts for each of the nine positions for each codon. In this study, we used the following criteria to define the significance of ribosome stalling: |RRT| ≥ 1.2, *P* ≤ 0.0001, and ≥ 300 available windows for a codon, to filter low quality sites. The A-, P-, and E-site on ribosome were defined previously as the position 6, 5, and 4, respectively [45].

### Identifying 5’extension and 3’read-through in translation

To identify 5’ extension in both strains, we followed the method in Gerashchenko *et al*. [46] with modifications: we first obtained the 180 bp (60 amino acids) upstream sequences for each orthologous pair in both strains, and then searched these sequences to see whether there was an in-frame start codon (ATG) in the upstream sequences, and no stop codon between this ATG and the predicted downstream start codon. We found 131 genes in both strains. The 5’ extension events were confirmed for these genes if ≥ 10 reads were observed in both mRNA and RFP, and these reads could cover ≥ 80% of the sequence of the extended region. For 3’ read-through identification, we searched for a stop codon (TAG, TAA, TGA) in the downstream sequences (180 bp from original stop codon) in both strains which resulted in 2,463 genes for further analysis. We used similar criteria (≥ 10 reads for both mRNA and RFP, and ≥ 80% sequences being covered) to confirm the 3’ read-through events. Both 5’ extension and 3’ read-through events were manually checked via IGV.

### Mitochondrial replacement

mtDNA in YJM789 was replaced using cytoduction [47]. First, the BY4742 strain was mated to a strain devoid of mtDNA, which carries the dominant *KAR1-1* mutation inhibiting nuclear fusion during zygote formation (strain MCC123 ρ^0^). Zygotes were then transferred to SC/leu^-^ medium to select for cells with an MCC123 nucleus and BY4742 mtDNA. This intermediate strain was further mated to the mtDNA-deficient (ρ^0^) strain of YJM789, and selected against uracil to obtain the combination of YJM789 nucleus and BY4742 mtDNA. We performed analogous experiments to replace the YJM789 mtDNA with that from itself as a control. All strains were checked for their genotypes with seven primer pairs distributed over the four chromosomes and mtDNA. Growth curves for all strains in both YPD and YPE media were monitored using BioTek (BioTek Instruments, Inc. USA), and transcriptome profiling was conducted using the protocol described above.

### Statistics

All statistics were performed using R version 2.15.2 (R Core Team 2012). FDRs for significant transcription/translation differences were calculated using the BH method incorporated in the p.adjust() function. The resampling method was implemented using the sample function in the ‘base’ package.

### Data access

The data from this study has been deposited in NCBI BioProject under the accession number: PRJNA281972. The genome sequence and annotation for *S*. *cerevisiae* used in this study was downloaded from Saccharomyces Genome Database (http://www.yeastgenome.org/, version R64-1-1), and sequence for YJM789 was obtained from Wei *et al*. [30].

## Results

### Transcriptome and translatome profiling in two *S*. *cerevisiae* strains

**Fig 1:**
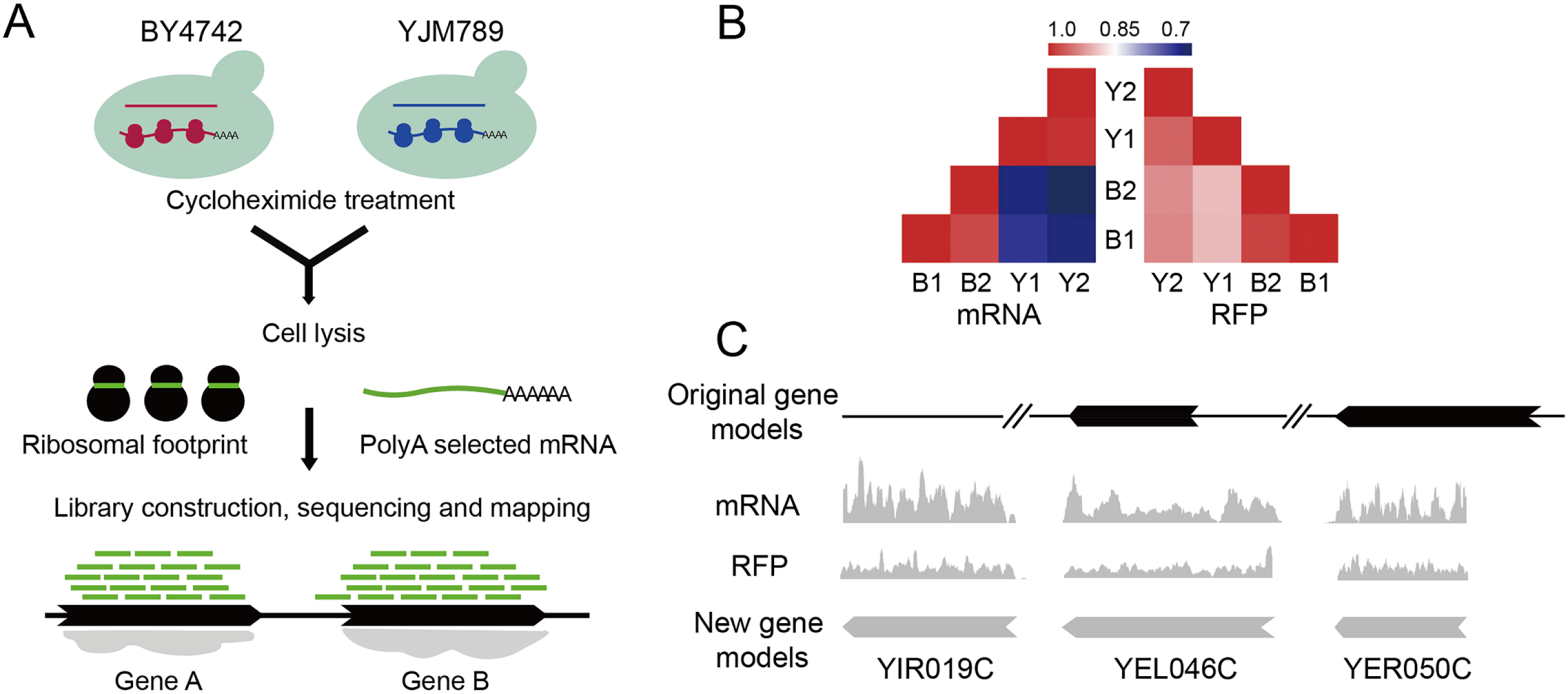
Transcriptome and translatome profiling for YJM789 and BY4742. **(A)** Workflow for the experimental procedures. The two yeast strains were cultured in YPD to log-phase. Cells were collected and treated with cycloheximide (CHX) for two minutes, and then divided into two aliquots, one for RNA-Seq and the other for ribosomal footprint profiling. Raw sequencing reads were cleaned and mapped to the yeast genomes for further analyses. **(B)** Spearman correlation coefficient (ρ) among mRNA and RFP data between biological replicates (indicated by numbers) and strains (indicated by letter, B: BY4742 and Y: YJM789). **(C)** Improving the YJM789 annotation using both mRNA and RFP data. Representative cases are depicted for three types of modifications: missing ORF (YIR019C), ORF extension (YEL046C) and ORF trimming (YER050C).

mRNA and RFP sequencing were conducted for a laboratory strain, BY4742, and a clinical strain, YJM789 (Fig 1A). Sequencing reads were mapped using an unbiased method with no more than two mismatches allowed [41]. A total of 7.6 ~ 10 and 2.2 ~ 3.6 million reads were mapped to ORFs for mRNA and RFP, respectively. The sequencing reads for each strain revealed good correlation between biological replicates (Spearman correlation coefficient: *ρ* ~ 0.98 for mRNA data, *ρ* ~ 0.99 for RFP data). The correlation for the sequencing reads between two strains indicated significant changes for the transcriptome and translatome (*ρ* ~ 0.72 and *ρ* ~ 0.95 for mRNA and RFP respectively) (Fig 1B).

The mRNA and RFP data enabled us to fine tune gene annotations in YJM789. The current gene models for S288c are almost complete, given the extensive research conducted on this strain. However, for YJM789, the annotation has yet to be improved. In Fig 1C, we depict three cases of the refined gene models, including ORF addition (YIR019C), ORF extension (YEL046C) and ORF trimming (YER050C). Altogether we were able to modify 85 gene models that were supported by both mRNA and RFP data in YJM789 (S1 Table).

### Strain differences in gene expression and translation

**Fig 2:**
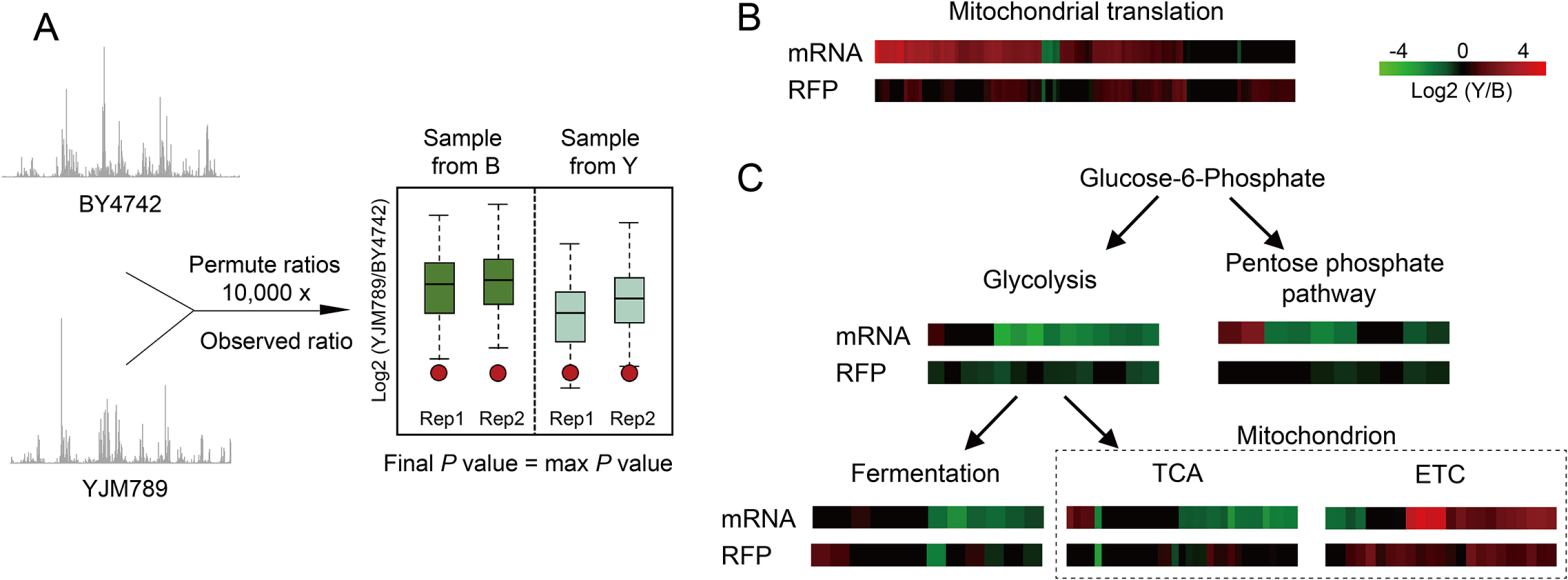
Strain differences in mRNA and RFP. **(A)** Diagram describing the resampling statistics. As introduced by Artieri and Fraser [13], for a given ortholog pair, we calculated the marginal nucleotide frequencies (π_B_ = [π_B_(A), π_B_(T), π_B_(G), π_B_(C)] for BY4742 and π_Y_ = [π_Y_(A), π_Y_(T), π_Y_(G), π_Y_(C)] for YJM789) and mappable lengths (L_B_ for BY4741 and L_Y_ for YJM789) for both genes, We began to generate the null ortholog pairs by resampling, with replacement, the base level counts from either BY4742 or YJM789 ortholog using the same length and nucleotide frequency from the orthologs (L_B_, π_B_ or L_Y_, π_Y_). The resampling was repeated 10,000 times to form the null distribution of log_2_(YJM789/BY4742) either from the BY4742 ortholog (green boxplot) or from the YJM789 ortholog (blue boxplot). The *P* value was obtained by comparing the observed log_2_ ratio to the null distributions, and the max *P* value from all four comparisons was assigned to that gene. Log_2_-ratio of YJM789 to BY4742 for genes involved in mitochondrial translation **(B)** and glucose metabolism **(C)**. The log_2_(Y/B) for genes with insignificant *P* values were manually set to zero in both (B) and (C). The dashed box shows the pathways involved in mitochondrial metabolisms.

To quantify strain differences in mRNA and RFP abundance, we excluded redundant or overlapping genes in both genomes, and retained those with only one ORF in each chromosome location. We furthermore filtered genes with fewer than 50 mapped reads, resulting in 5,301 and 3,663 ones to be investigated for transcriptome and translatome, respectively. It is known that Illumina sequencing can be biased toward locally varying GC content [48]. However, the mapped RFP reads, as shown in the S1 Fig, were preferentially enriched in A/T, especially on the first base. This finding is consistent with a recent report [49], and may be in part due to ribosome aggregation in the start codon or translation pausing in specific sites [50]. Notably, because the sequencing bias and read distribution are quite different between mRNA and RFP, the conventional statistical models for testing differential gene expression might lead to bias in quantifying differential gene translation [50]. A resampling method, which took into account both uneven read distributions and gene length variations to identify genes with differential expression and translation, has been developed recently [13, 51]. We applied this method in our data analysis (Fig 2A).

Overall, at a false discovery rate (FDR) ≤ 5%, 67% (n = 3,570) and 45% (n = 1,632) of all genes showed significant differences in mRNA and RFP between strains, respectively. Functional analysis of differentially regulated genes using GO [52] revealed that multiple functional categories show significant differences in either mRNA or RFP level (S2 Table). Interestingly, only genes involved in mitochondrial functions show consistent up-regulation in YJM789 for both mRNA and RFP levels (Table 1), whereas genes related with amino acid biosynthesis were more active in BY4742 for the two levels (S2 Table). It has been shown that the laboratory strains have more auxotrophies in amino acid biosynthesis [7], so we were particularly interested in investigating the regulation divergence of mitochondrial functions between these two strains in the following research. Among all categories related with mitochondrial functions, only two basic biological processes—mitochondrial translation (fold enrichment = 2.19 for mRNA and 3.75 for RFP, Fig 2B) and electron transport chain (ETC, fold enrichment = 1.73 for mRNA and 2.89 for RFP, Fig 2C), showed regulatory differences that are statistically significant at both mRNA and RFP levels. In contrast to the ETC, the cytosolic part of the carbohydrate metabolism pathway showed decreased or balanced mRNA or RFP abundance in YJM789 (Fig 2C).

**Table 1:** Enrichment of up-regulated genes in YJM789 that are related with mitochondrial functions.

Cellular component	Fold enrichment		*P* value		Adjusted *P* value	
	mRNA	RFP	mRNA	RFP	mRNA	RFP
Mitochondrial ribosome (GO:0005761)	2.28	3.61	1.39E-15	9.16E-26	2.80E-13	1.39E-23
Mitochondrial respiratory chain (GO:0005746)	2.06	3.86	9.89E-4	4.55E-9	1.90E-2	1.73E-7
Mitochondrion (GO:0005739)	1.11	1.69	1.09E-3	1.66E-29	2.03E-2	3.79E-27

### Heme-activated proteins and mRNA divergence for the ETC genes

**Fig 3:**
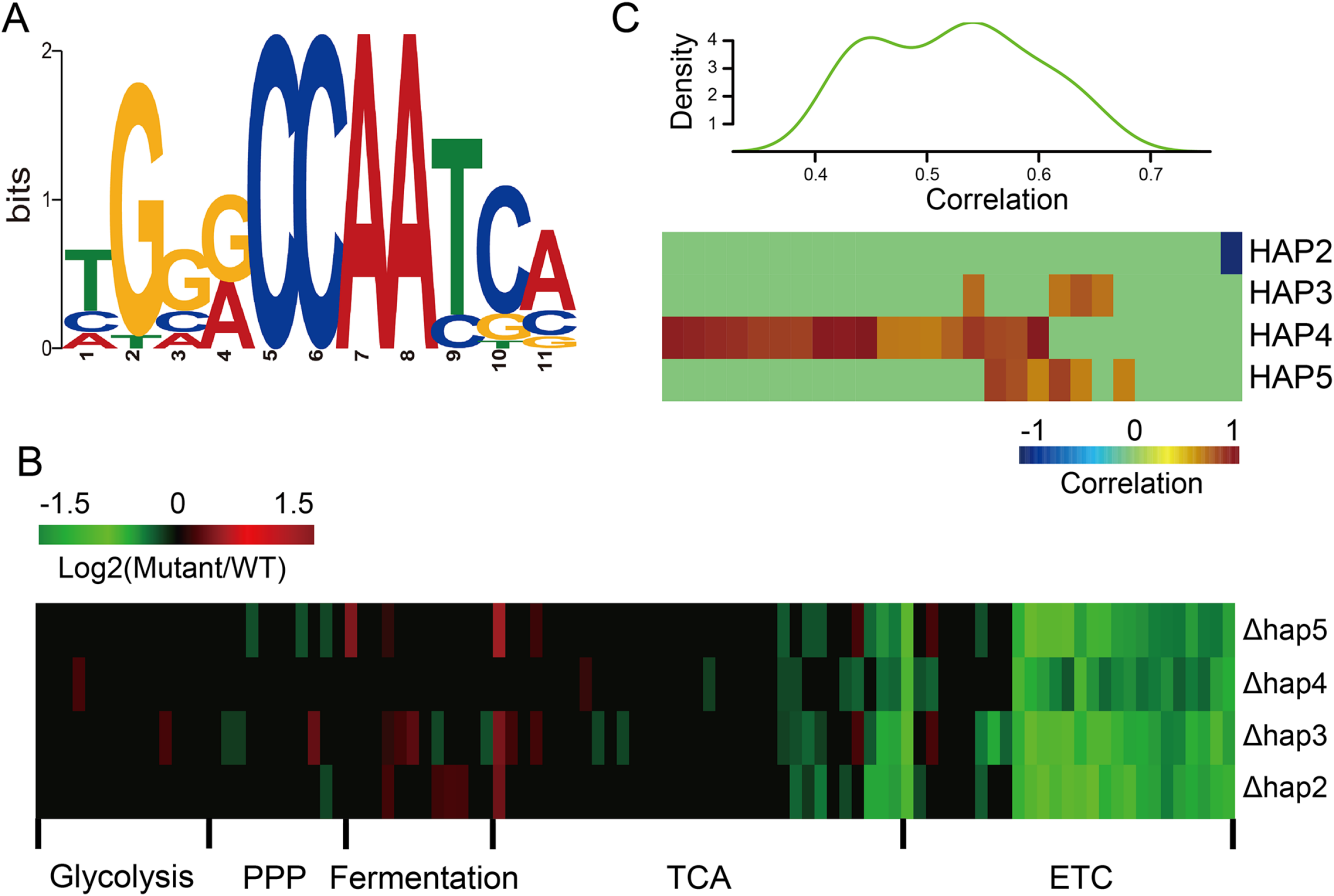
*HAP4* and mRNA divergence for ETC genes. **(A)** The *HAP4* motif is enriched in the promoter regions of the ETC genes. **(B)** Log_2_ (expression change) for genes involved in glucose metabolisms for the *HAP2*/*3*/*4*/*5* gene deletion mutants. Data was adopted from Kemmeren *et al*. [56]. Genes with insignificant *P* values were manually set to zero. **(C)** Correlation coefficient of gene expression between each of the HAP complex components and the ETC genes among 22 yeast strains (upper panel: overall distribution of the correlation coefficients; lower panel: individual correlation coefficients). The RNA-Seq data was from Skelly *et al*. [19].

To reveal possible regulatory factors for genes involved in ETC and mitochondrial translation, we searched for overrepresented motifs in the 1 kb upstream and downstream sequences of genes in both categories using MEME [44]. A motif with the core consensus sequence CCAAT was significantly enriched in ETC genes (Fig 3A). Comparing this motif to the known motif collections in TOMTOM [53], we observed high similarity with the binding site of the heme-activated protein (HAP) complex (*P* = 2.1E-4, Fig 3A). The HAP complex (*HAP2*/*3*/*4*/*5*) was previously reported to play a major role in orchestrating the transcription of genes involved in respiration [54]. *HAP2/3/5* bind to DNA and are constitutively expressed, while *HAP4* regulates the activity of the complex and is repressed by glucose [55]. To investigate whether expression changes for the ETC genes between two strains are associated with the HAP complex, we reanalyzed the global dynamic gene expression profiles for the HAP deletion mutants from a recently published work, where the dynamics of gene expression in over 1,400 deletion mutants were profiled [56]. Results indicated that all HAP components directly regulate expression of most genes in the ETC, but only a few genes in other respiration and cytosolic metabolism related processes (Fig 3B).

The expression of the *HAP2*/*3*/*4* genes was significantly different between the two studied strains, with *HAP2* down-regulated and *HAP3*/*4* up-regulated in YJM789. However, only *HAP4* was up-regulated at the RFP level in YJM789, indicating that Hap4p up-regulation might be the cause of the observed expression differences for the ETC genes between the two studied strains. Indeed, this conclusion is supported by reanalyzing another recently published work, in which the transcriptome for 22 *S*. *cerevisiae* strains (YJM789 was not included) were profiled [19]. As shown in Fig 3C, 85% of ETC genes were positively correlated (*P* ≤ 0.05) with expression of one or more HAP complex components. Among these genes, 61% ETC genes were only correlated with expression of the *HAP4* gene, indicating that the dynamic regulation of the *HAP4* gene likely underlies the transcriptional differences within ETC genes among different yeast strains.

It should be noted that, the *HAP1* also regulates the genes involved in respiration and sterol biosynthesis [57]. However, the S288c and its derivate carry a Ty1 insertion in this gene, which makes it a null allele [58]. We proved that the functionality of *HAP1* in BY4741 (null allele) and YJM789 (functional allele) causes little, if any, impact on ETC gene expression. Firstly, other strains with functional *HAP1* gene also showed highly diverged mitochondrial functions (S2 Fig) [19]. Secondly, most ETC genes are not directly regulated by Hap1p, only 37% ETC genes are expressed in anti-correlation (*r* = -0.63~-0.45, *P* ≤ 5%) with *HAP1* among different yeast strains.

### *PUF3* and mRNA/RFP divergence for genes in mitochondrial translation

**Fig 4:**
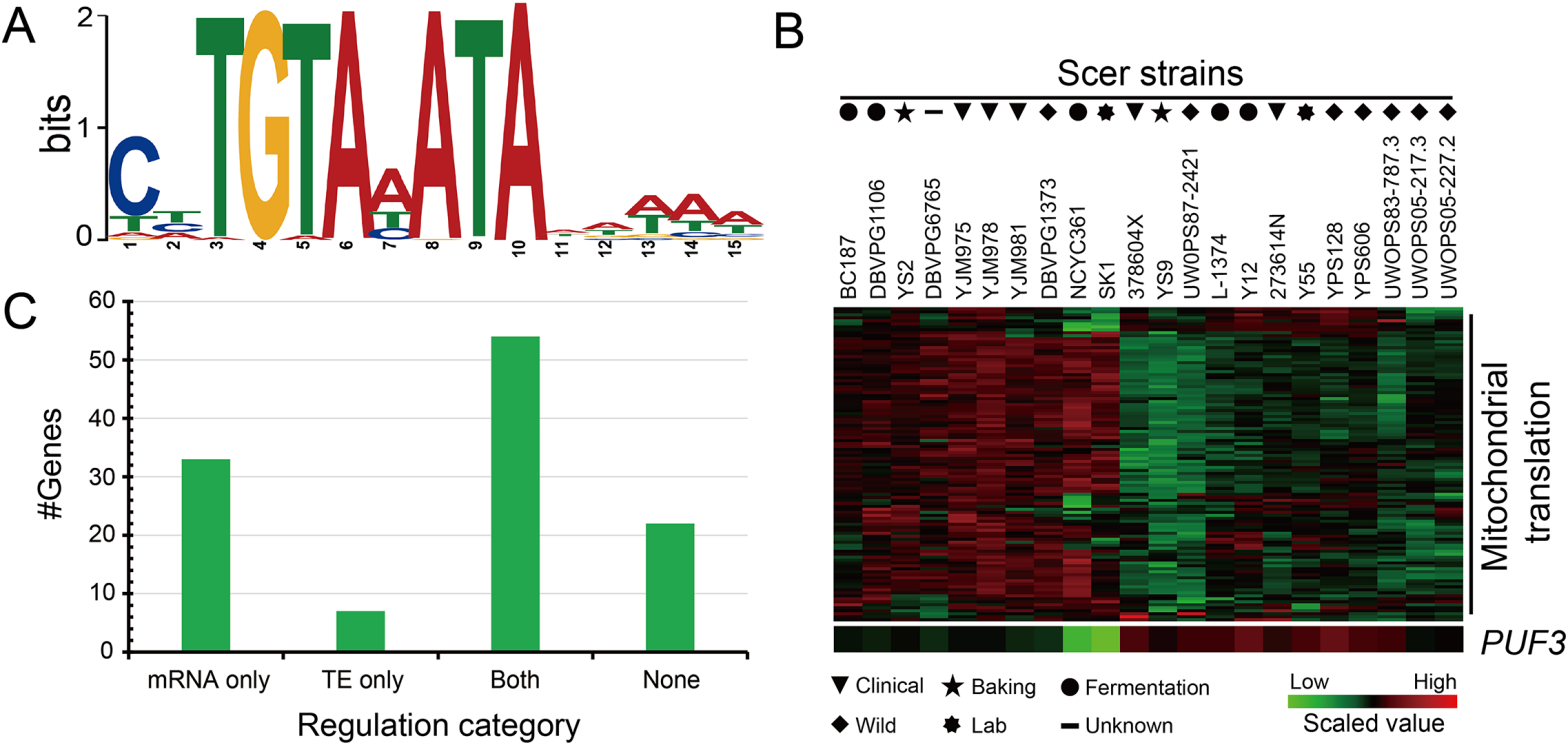
*PUF3* and mRNA/RFP divergence for mitochondrial translation related genes. **(A**) The *PUF3* motif is enriched in 3’ UTRs of genes in mitochondrial translation. **(B)** The expression of mitochondrial translation genes and *PUF3* from the 22 yeast strains. Data was from Skelly *et al*. [19]. **(C)** Categorizing the regulatory effects of *PUF3* on mitochondrial translation genes. Genes were classified into four categories based on their significance (FDR ≤ 5%) in mRNA or translation efficiency (TE).

We found no enriched motifs in the upstream promoter sequences of genes involved in mitochondrial translation. Instead, we discovered a core TGTAHATA motif in the downstream sequences of all investigated genes (Fig 4A). 81% of its occurrences were located within 100 bp from the stop codon. This motif was reported to be the binding site for an RNA-binding protein, *PUF3* [59]. The enrichment of the *PUF3* binding motif within mitochondrial translation related genes in this study, agrees with the known role of *PUF3* in regulating mitochondrial functions [59, 60], and suggests divergent activity of *PUF3* between two strains. Indeed, the mRNA [log2 (fold change) = -1.83] and RFP [log2 (fold change) = -0.42] levels of the *PUF3* gene in YJM789 were both significantly lower than in BY4742. This observation was also confirmed by the data from the aforementioned 22 *S*. *cerevisiae* strains, which showed that a majority of genes involved in mitochondrial translation were anti-correlated (*P* ≤ 0.05) with the abundance of the *PUF3* gene (the correlation coefficient ranged from -0.90 to -0.43, Fig 4B).

It has been proposed that Puf3p functions in both promoting mRNA decay and affecting mRNA translation [61]. In this study, we found that 75% (87/116) of mitochondrial translation related genes were significantly different between two strains at the mRNA level, and 65% (75/116) showed significant changes at the RFP level. To explore how many genes are regulated at post-transcriptional level, we calculated the translation efficiency (TE) by dividing the counts in RFP by that in the corresponding mRNA (RFP/mRNA) of the same gene. Altogether, 28.4% (33/116) genes showed changes only at the mRNA level, and 46.6% (54/116) genes displayed divergence at both the mRNA and TE levels. We also identified seven genes that diverged at the TE level alone (Fig 4C). We note that the mRNA and RFP were profiled at the exponential growth condition, regulation of which may differ from other growth conditions. Due to the sophisticated roles reported on Puf3p, these results suggested that Puf3p may function by influencing both mRNA stability and translation, and the impact on mRNA stability may contribute larger than on translation, leading to strain differences in gene regulation.

### Compatible mitochondrial and nuclear genomes between two strains

Mitochondrial function is determined by proteins encoded by both nuclear DNA and mitochondrial DNA. We further investigated whether the mitochondrial genome in YJM789 has unique changes that might have adapted to the likely enhanced mitochondrial activity in this background. The two mitochondrial DNA (mtDNA) genomes differ in size with the YJM789 mtDNA 435 bp longer than the BY4742 mtDNA. Comparing between the 10 mtDNA encoded proteins, we identified more than 63 SNPs and two indels distributed over five genes (*COX1*, *ATP6*, *COB1*, *VAR1* and *COX2*), leading to 32 amino acid changes (S3 Table). No SNPs were found for all 24 mtDNA encoded tRNA genes.

**Fig 5:**
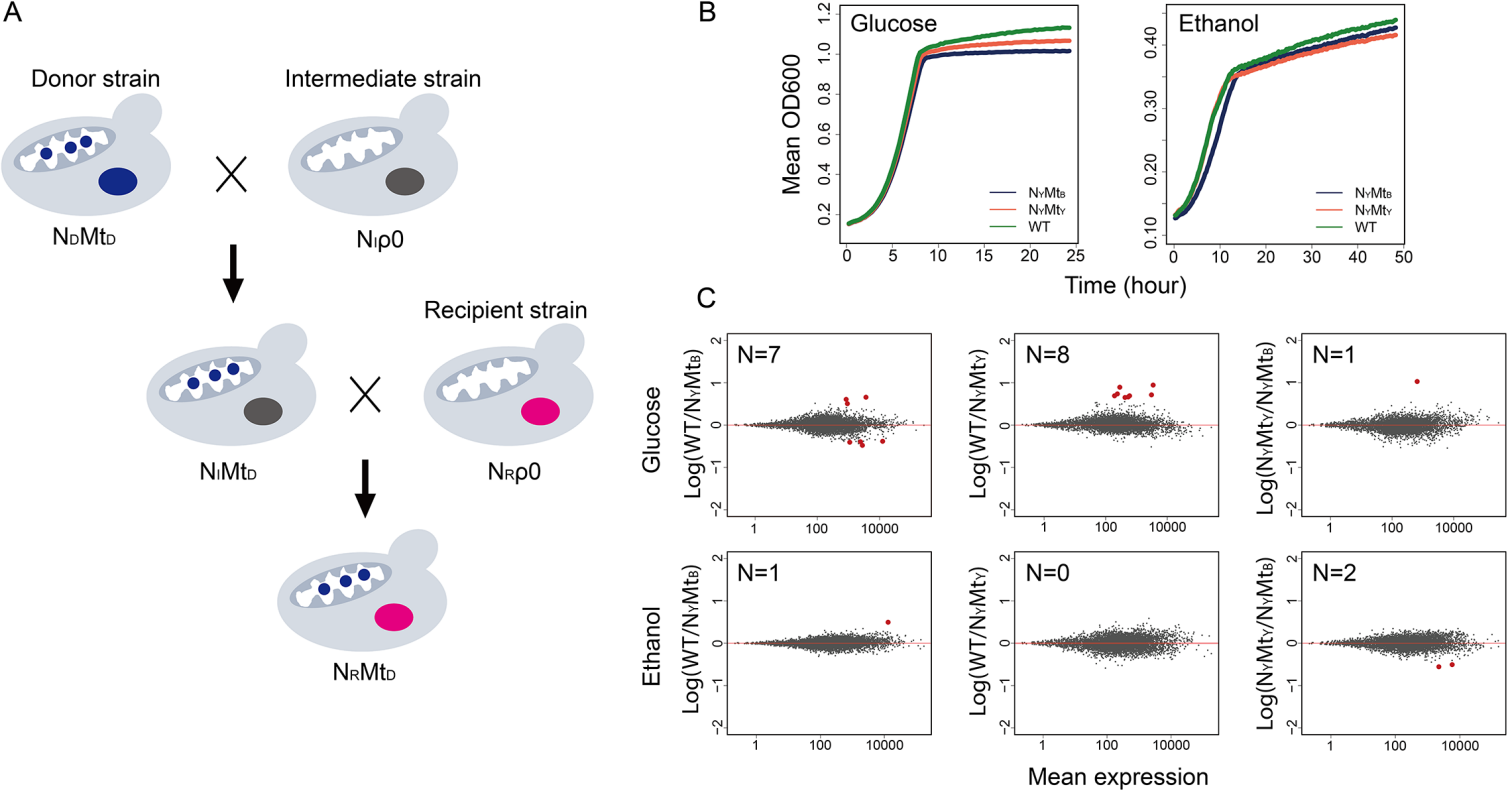
Compatible mito-nuclear genomes in two yeast strains. **(A)** Cytoduction was used to construct mtDNA replacement strains in the YJM789 genetic background [47]. Successful replacements underwent two rounds of mating. In the first round, an mtDNA donor strain was mated with an intermediate strain, the *KAR1-1* mutant, which is devoid of mtDNA. Cells with a nuclear background from *KAR1-1* and mtDNA from the donor strain were selected for the next round of mating with the recipient strain, which is also mtDNA deficient. Finally, cells with nuclear DNA from the recipient strain and mtDNA from the donor strain were selected and confirmed for their genotypes using several markers from different chromosomes. **(B)** Yeast cells were cultured in either glucose or ethanol growth medium starting at OD600 = 0.1. Three strains were used for the experiment, WT: YJM789 wild type, N_Y_Mt_Y_: YJM789 nuclear genome + YJM789 mtDNA; N_Y_Mt_B_: YJM789 nuclear genome + BY4742 mtDNA. The shown values were the average from three independent colonies for each strain. **(C)** Transcriptome profiling for each strain cultured in glucose or ethanol medium. Red dots show genes with significant expression changes, with the number of red dots indicated for each plot. The cutoff defining significance in these figures is FDR ≤ 10%.

To explore whether these structural variations could have any functional impact, we replaced the YJM789 mtDNA with that from BY4742 using the cytoduction method [47] (Fig 5A), and investigated the impact of mtDNA replacement on organism growth and gene expression. We grew the WT and mtDNA replaced YJM789 strains in both fermentative (glucose) and respiratory (ethanol) growth media, and monitored their growth every 15 minutes. The growth of the WT and the mtDNA replaced strains (N_Y_Mt_Y_: YJM789 nuclear DNA + YJM789 mtDNA, N_Y_Mt_B_: YJM789 nuclear DNA +BY4742 mtDNA) were similar in either growth condition (Fig 5B).

We also conducted RNA-Seq for the WT and mtDNA replaced strains. For gene expression analysis, we first set the cutoff of FDR ≤ 5%, which yielded only fewer than 5 genes that were significantly different in all three pairwise comparisons in the glucose growth medium, and only one gene in the ethanol growth medium (N_Y_Mt_Y_ vs N_Y_Mt_B_). Increasing the cutoff to FDR ≤ 10% only resulted in few more genes showing differential expression among genetic backgrounds in both glucose and ethanol growth media, respectively (Fig 5C). Furthermore, the differentially expressed genes were not consistent among different pairwise comparisons. These data suggested that mtDNA between BY4742 and YJM789 are functionally interchangeable under the tested culture conditions, and that the mtDNA genome does not contribute to the observed gene regulation differences between the two studied strains.

### Strain differences in other features revealed by RFP

**Fig 6:**
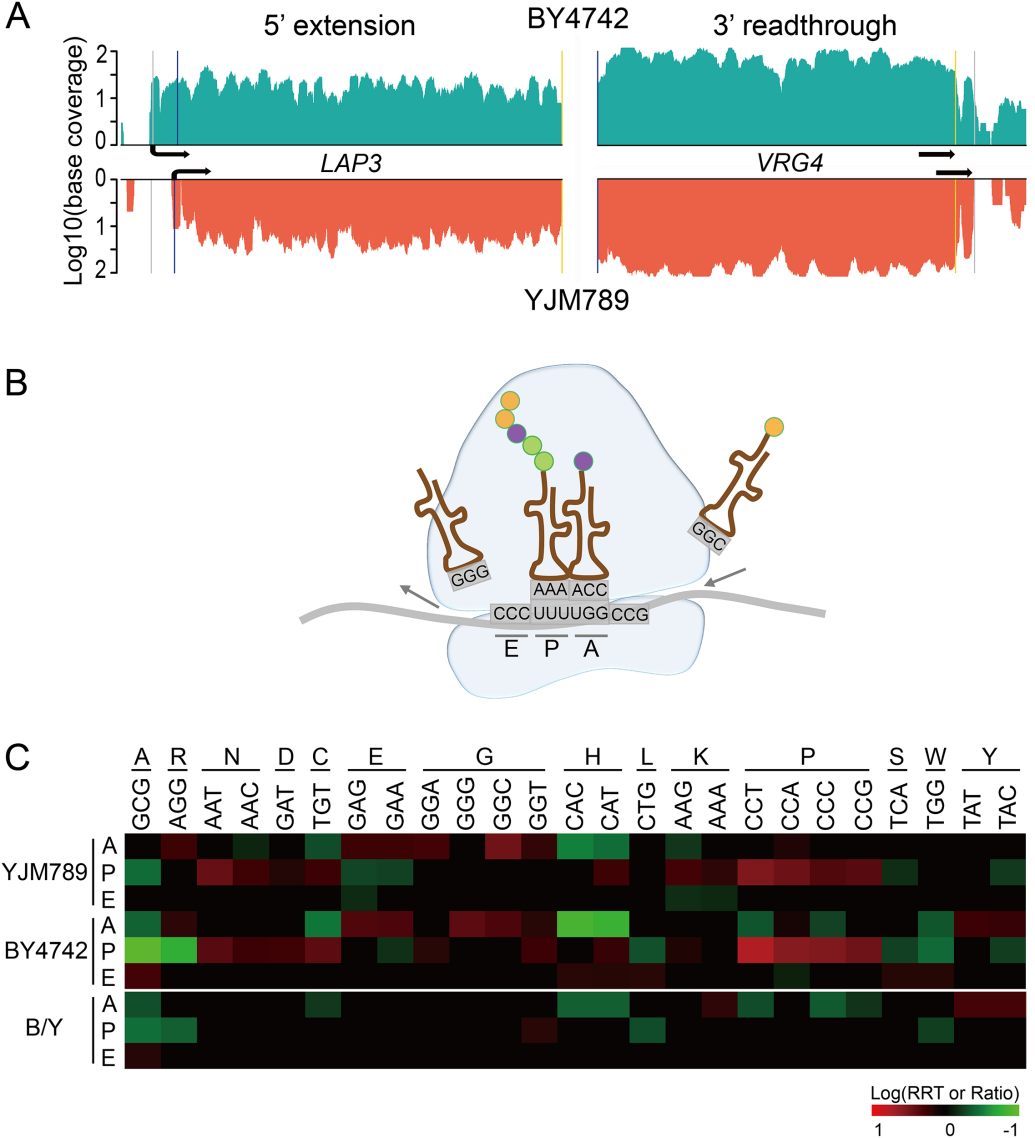
5’-extension, 3’-readthrough and ribosome stalling for the two strains. **(A)** Two examples for 5’ extension and 3’ read-through in two strains. The blue and yellow lines indicate the positions of the original start and stop codons, respectively. The gray line shows the extended position of either the start or stop codons. Arrows indicate the ORF directions. **(B)** A cartoon diagram depicting the A-、P- and E- sites in the ribosome. The A-site is occupied by aminoacyl-tRNA, which functions as the acceptor for the growing protein during peptide bond formation. The P-site is occupied by the peptidyl-tRNA, the tRNA carrying the growing peptide chain. The E-site contains the deacylated tRNA on transit out from ribosome. **(C)** Ribosome residence time (RRT) for the 25 selected codons on A-, P- or E- sites of ribosome in two strains. Positions for those codons with insignificant *P* values were set to zero (RRT = 1). All other codons are not included because they do not show any significant stalling (defined as RRT ≥ 1.2 and *P* ≤ 0.0001) in either background. Codons were shown on the columns, with the respective amino acids indicated above. To compare the RRT for selected codons between strains, the ratio of RRT for two strains (B/Y) was calculated for each permutation. The observed RRT ratio was then compared with the permutated null ratios to obtain the *P* value. The criteria defining significance are the same as for ribosome stalling.

Owing to the unique advantages of RFP, we were able to explore genome wide differences for the features other than abundance. For example, we investigated peptide diversity by 5’ end extension or 3’ end read-through, which have been proposed as possible mechanisms to accelerate environmental adaptation [46]. Using both mRNA and RFP data, we found 4 genes with 5’ extension (Fig 6A, S4 Table). Of these, two genes (*LAP3* and *RSM18*) were only found in BY4742. *LAP3*, a cysteine aminopeptidase with homocysteine-thiolactonase activity [62], was extended by 30 codons just upstream of the original start codon in BY4742. Comparing the sequences of the 30 codons between the two strains, we found only one C to T mutation at the -22 upstream codon, converting ‘CGG’ to ‘TGG’ in YJM789. For *RSM18*, a mitochondrial ribosomal protein gene of the small subunit [63], 13 codons were added to the 5’ terminus in BY4742, whereas a stop codon ‘TAG’ is present before the start codon in YJM789, which may prevent the extension in that strain. In addition to 5’ extension, we also discovered 34 candidate genes with 3’ read-through events (Fig 6A and S5 Table) in both strains. Of these, four cases (*MBF1*, *RBG1*, *CBF5* and *VRG4*) were also observed in other yeast species in a previous study [13]. A BLAST analysis of the 5’ end extension or 3’ end read-through peptides against closely related yeast species indicated that they were not evolutionarily conserved.

The RFP data also provides an opportunity to systematically investigate ribosome movement in cells. We compared the overall ribosomal status in two strains by calculating the ribosome residence time (RRT) for all 61 codons at the A-site (occupied by aminoacyl-tRNA), P-site (occupied by peptidyl-tRNA) and E-site (occupied by deacylated tRNA on transit out from ribosome, Fig 6B) [45]. Consistent with previous reports [45, 49], all four codons (CCA, CCT, CCC, CCG) encoding proline showed significant translation stalling at the P-site in both strains (Fig 6C, panels YJM789 and BY4742). We also discovered other codons with over- or under-represented ribosome occupancy (Fig 6C, panels YJM789 and BY4742). For example, codons for glutamic acid (GAG and GAA) and glycine (GGC and GGT) were consistently enriched on the A-site, whereas codons for histidine (CAC and CAT) were under-represented on the A-site for both species. The overall selected ribosome occupancy in both strains is statistically indistinguishable for codons on E-site (Fig 6C, panel B/Y). Oppositely, some codons showed distinctive occupancy on either A-site (e.g. TAT and TAC for tyrosine) or P-site (e.g. GGT for glycine) between the two strains (Fig 6C, panel B/Y). The underlying reasons for the differential ribosome movement and frequency among yeast strains warrant further investigation.

## Discussion

### Divergence of gene expression and translation between strains

We found significantly more mRNA than RFP changes between the two strains (67% vs 45%), indicating that the translatome might be more stable than the transcriptome during evolution, a conclusion that has been shown previously from our research and other groups [5, 9, 37]. In this study, we further discovered that the divergent regulation of mitochondrial functions between two strains is indeed more significant at the translational level than the transcriptional level (Table 1). Genes in mitochondrial translation and ETC showed both higher transcription and translation in YJM789 than in BY4742. It is not appropriate to conclude the direction of mitochondrial evolution between the two strains from the current comparison, because both strains might change due to their unique environmental niche. When reanalyzing the transcriptome data from the aforementioned 22 yeast strains from diverse sources [19], we found that both the wild and lab strains are also diverged in the expression of mitochondrial translation genes within each groups, confirming our conclusion that yeast strains generally have diverged expression for genes related with mitochondrial functions, in particular for genes involved in mitochondrial translation and carbohydrate metabolic processes (S2 Fig).

YJM789 has been used as a model for understanding traits associated with pathogenic fungi [31, 33]. We investigated whether the elevated expression of genes functioning in mitochondria are associated with the possible pathogenic life style in YJM789. As shown in S2 Fig, the divergence of mitochondrial functions was not correlated with their pathogenicity, indicating that a relatively enhanced mitochondrial function is neither necessary nor sufficient for fungal pathogenicity. Nevertheless, whether the functional changes in mitochondria observed in this study could underlie certain traits in YJM789 needs to be further investigated.

### Coordinated regulation of the *HAP4* and *PUF3* network

We revealed that *HAP4* transcriptional regulation and *PUF3* post-transcriptional regulation were likely responsible for changes in gene regulation of the ETC and mitochondrial translation, respectively. The direction of change for both networks is consistent with an increased mitochondrial function in YJM789. To explore whether *HAP4* and *PUF3* could be co-regulated by a common factor, we compared the microarray data for over 1,400 yeast mutants [56]. Altogether there are 344 gene deletions can influence the *HAP4* expression, a number much larger than that of the other HAP components (158~217). Among these 344 genes, 85 showed significant expression correlation (positive or negative) with *HAP4* in the aforementioned 22 yeast strains (*P* ≤ 0.05, S6 Table). In contrast, only 11 gene deletions affect *PUF3* expression. Among them, *SWI6* is the only gene that was anti-correlated with *PUF3* (*r* = -0.54, *P* = 0.01006) among the 22 yeast strains (S3 Fig). *SWI6* is a subunit of the *SWI6*/*SWI4* complex, which activates the G1/S transition in the cell cycle. Interestingly, *SWI4* gene expression is positively correlated with *HAP4* among the 22 yeast strains (*r* = 0.46, *P* = 0.03087). Collectively, these data indicates that the *SWI4*/*6* complex could be co-regulated with both the *HAP4* and *PUF3* genes, which might play an important role in linking cell cycle regulation with mitochondrial activity. It has been shown that cellular metabolism is tightly coupled with cell cycle regulation [64, 65]. Previous work in *Drosophila* also showed that disruption of the ETC complex I retarded the cell cycle during the G1/S transition [66]. Whether *SWI4*/*6* directly regulates the *HAP4* and *PUF3* genes remains to be studied.

There might be some other factors contributing to our observation. For example, genetic variation in a major transcriptional regulator MKT1 (D30G) has been shown underlying changes in some mitochondria-related phenotypes between yeast strains [67–69]. Smith and Kruglyak [67] surveyed gene-environment interaction using yeast gene expression under glucose and ethanol growth conditions. MKT1 was identified as a candidate for the proposed polymorphism cluster for the gene and environment interactions. However, in the glucose environment, which is the same condition used in our study, no mitochondrial related genes/functions were enriched in the genes affected by the MKT1 alleles (S1 Table in the original publication). Dimitrov et al. [68] used phenotype QTL mapping to successfully identify alleles contributing to the petite frequency in yeast. MKT1 was one of several loci identified. It is important to note that the petite formation reflects mitochondrial genome integrity but not the expression of genes functioning in mitochondria. Brion et al [69] did both phenotype and expression QTL mapping in yeast, and identified a hotspot of 101 eQTLs on MKT1. However, the study used simulated grape must as the growth condition. Interestingly, the amino acid at position 30 of MKT1 is D in BY4742 and G in YJM789, respectively. Whether this variation is related with our observation in this study needs to be investigated in the future.

### Mito-nuclear interactions between the two yeast strains

Previous work on mito-nuclear interactions in several organisms has reported that interfering the standing mito-nuclear interplay may cause fatal problems to their viability or reproduction [26, 27, 70]. In yeast, disturbing the naturally occurring mito-nuclear genome combinations can provide growth advantages, indicating a significant amount of epistatic interactions between mitochondrial and nuclear genomes among yeast strains [23]. Interestingly, in the present work, replacing mtDNA in the YJM789 background did not affect its growth rate in both fermentative and respiratory media, and the overall gene expression did not change either, providing evidence for compatible mito-nuclear interactions between these two studied strains. However, it needs to be noted that the mito-nuclear genomes interaction has only been observed in one direction. Furthermore, because the mitochondrion is often involved in stress responses, and plays critical roles in signaling, it remains interesting to investigate whether the impact of mito-nuclear interactions would be revealed under other specific growth conditions.

## Supporting Information

S1 Fig The A/T rich sequencing bias for ribosomal footprint profiling. (TIF)

S2 Fig Expression divergence of genes involved in mitochondrial translation and glucose metabolism in 22 yeast strains. (TIF)

S3 Fig Negatively correlated expression of *PUF3* and *SWI6* across yeast strains. (TIF)

S1 Table The modified gene models in YJM789. (XLSX)

S2 Table Enrichment of biological processes for genes with differential regulation in mRNA and RPF. (XLSX)

S3 Table Genomic variations between mtDNA in two strains. (XLSX)

S4 Table The genes with 5’ extension identified in the two strains. (XLSX)

S5 Table The genes with 3’ read-through identified in the two strains. (XLSX)

S6 Table Gene expression correlation between *HAP4* and its possible regulators. (XLSX)
