# Supplementary figures and images for: Coordinated Evolution of Transcriptional and Post-Transcriptional Regulation for Mitochondrial Functions in Yeast Strains
Source: PLoS One. 2016 Apr 14;11(4):e0153523. doi: 10.1371/journal.pone.0153523 (PMC4831757; doi:10.1371/journal.pone.0153523)

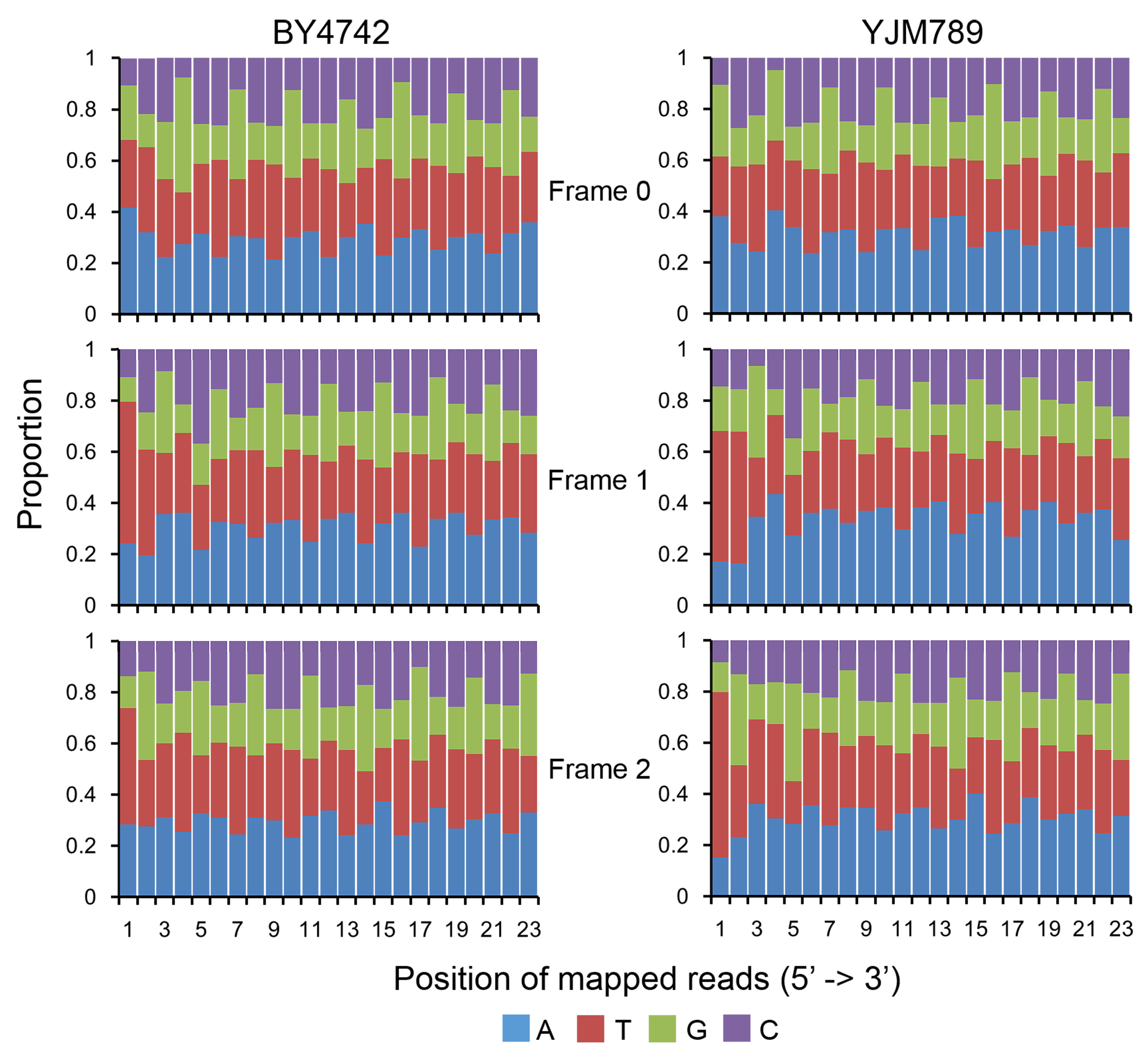

Supplement: S1 Fig — (TIF) [file pone.0153523.s001.tif]

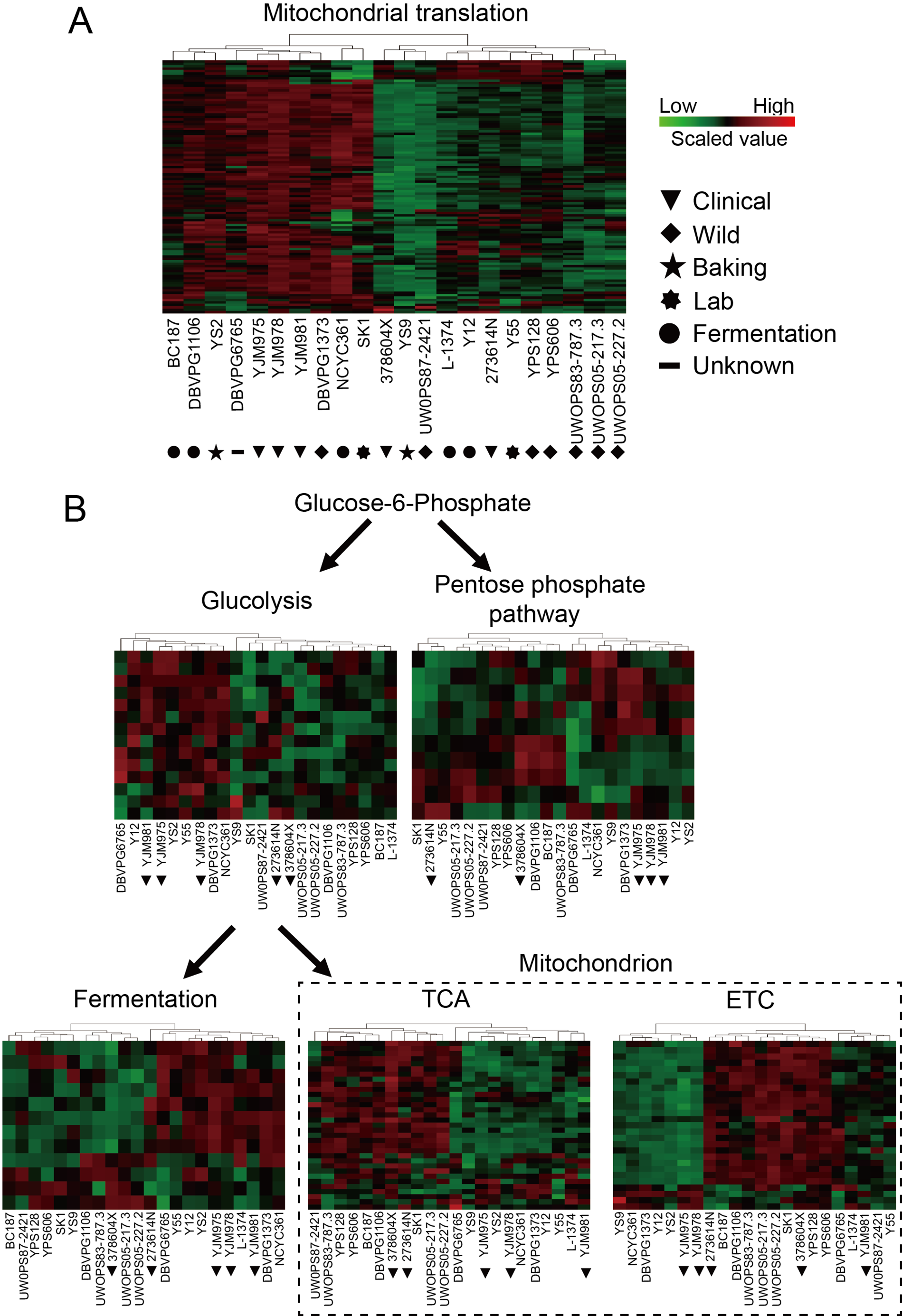

Supplement: S2 Fig — (TIF) [file pone.0153523.s002.tif]

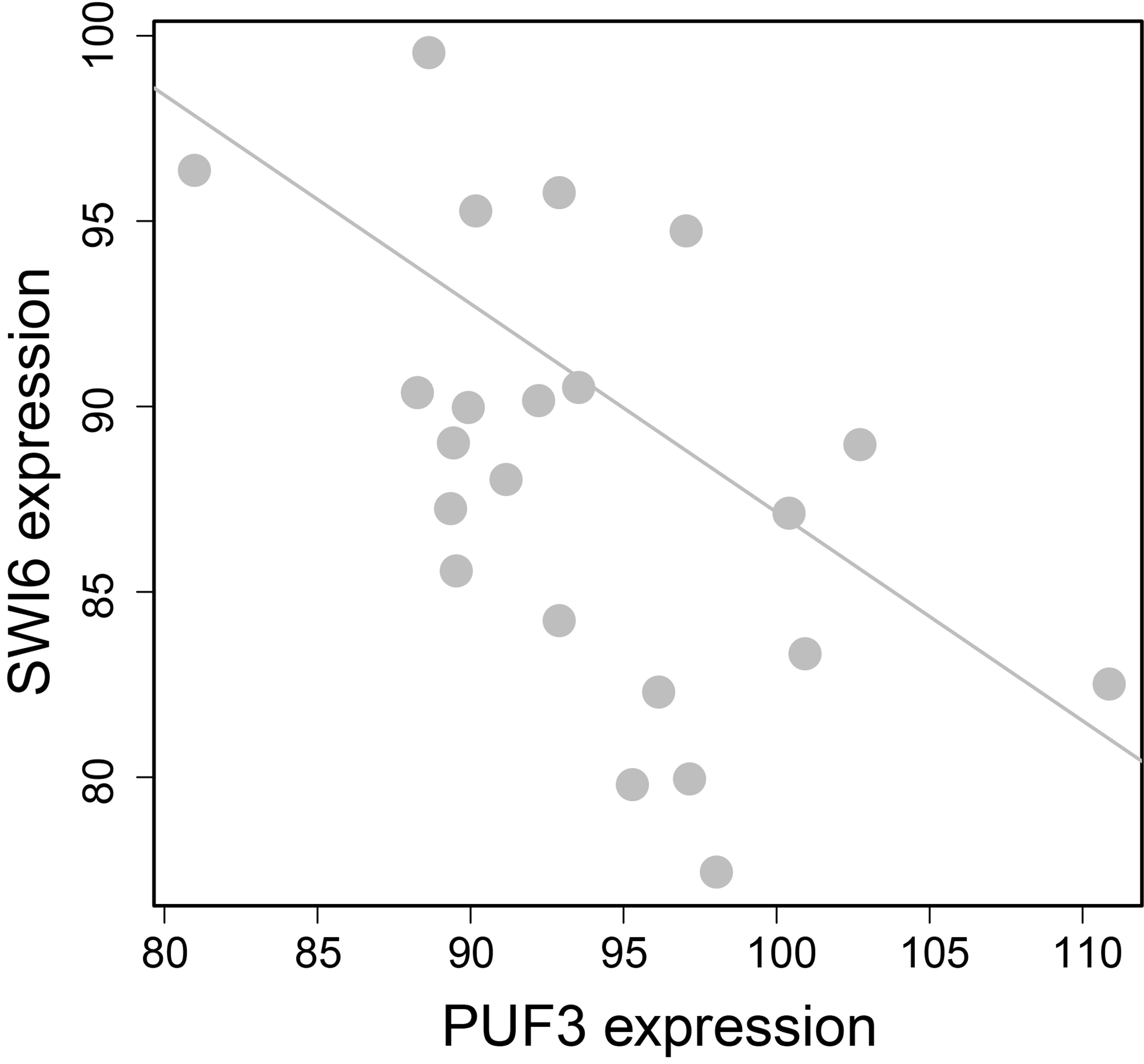

Supplement: S3 Fig — (TIF) [file pone.0153523.s003.tif]
